# Ras: structural details to guide direct targeting

**DOI:** 10.18632/aging.100754

**Published:** 2015-05-30

**Authors:** Carla Mattos

**Affiliations:** Department of Chemistry and Chemical Biology, Northeastern University, Boston, MA 02115, USA

Ras proteins are small GTPases, found in distinct isoforms, HRas, KRas (KRas4A and KRas4B) and NRas. These proteins are mutated in about 20% of all human cancers and are thus important targets for drug discovery [1]. Major efforts over the last 20 years have not yielded drugs, promoting the notion that Ras is undruggable [2]. Recent advances in the structural biology of Ras provide new venues to be explored.

The crystal structure of Ras has been known for over 25 years. Structural rearrangements that occur in the transition from Ras-GTP to Ras-GDP have been well studied, as GTP hydrolysis is at the center of how signaling through Ras is turned off. By the year 2000 we had the structures of several oncogenic mutants and the Ras/GAP complex revealing the transition state of the GAP-catalyzed reaction. Whether intrinsic or GAP-catalyzed, the hydrolysis reaction has traditionally been studied from the perspective of the active site without consideration of long-rage, global effects on catalysis. More recent focus has turned to the involvement of dynamics and conformational states of Ras-GTP with intrinsic hydrolysis on Ras. The study of allosteric modulation of the active site has revealed that in addition to known catalytic residues, switch II, helix 3, loop 7 and helix 4 may modulate hydrolysis, particularly in the complex with Raf [3]. These structural elements are affected by Ca2+ binding at an allosteric site remote from the nucleotide-binding pocket and thought to interact with the membrane [4]. Thus, within the context of allosteric modulation, hydrolysis of GTP on Ras is a global endeavor. However, given the existing framework of hydrolysis as being local to the active site, we have constructed an understanding of the effects of oncogenic mutants as a local phenomenon as well, focusing on the active site to study how it is perturbed in these mutants. In the context of global involvement of the Ras structure in catalysis, we must consider global effects due to oncogenic mutations. Indeed, our group has recently shown that the highly oncogenic Q61L mutant affects not only distal portions of Ras, but also of Raf-RBD in the complex [5]. Remarkably, the binding of Raf-RBD to wild type HRas promotes a decrease in flexibility of residues that coordinate Ca2+ at the remote allosteric site, adding to our proposed mechanism that interaction with the membrane primes the site for calcium binding to enhance intrinsic hydrolysis in the complex with Raf [3]. Thus, our current hypothesis is that Raf-RBD works synergistically with the membrane to render the allosteric site receptive to Ca2+ binding, triggering GAP-independent hydrolysis to turn off signaling through Ras/Raf.

The global effects of the Ras structure on hydrolysis are mediated by networks of communication between the active site and distant structural elements of Ras that interact with the membrane [6]. The detailed features of these networks and how they are impaired by oncogenic mutations can provide important clues for unexplored drug target sites on Ras [2]. Several sites identified as hot spots of protein-ligand interactions [7], in addition to intercepting the networks of communication across the Ras catalytic domain, are located in areas that have the most differences between H, K and NRas [4]. Thus, while the intramolecular networks of communication are likely common to the three Ras isoforms, they may be affected differently by ligand binding to each one. As we move forward it will be important to determine whether a given mutation affects the isoforms in different ways, particularly in terms of global dynamics and conformational states associated with hydrolysis in the presence of Raf. It is increasingly clear that Ras mutants do not collectively constitute a general target that can be addressed with a single approach. Instead each specific mutant of a specific isoform will have to be addressed as a separate target within the context of how the mutation affects the wild type features essential to Ras function.
